# Estimation of esophageal involvement length in esophagogastric junction tumors using PET-CT

**DOI:** 10.1007/s10388-026-01183-6

**Published:** 2026-01-28

**Authors:** Shinnosuke Nagano, Yukinori Kurokawa, Takaomi Hagi, Takuro Saito, Tsuyoshi Takahashi, Shigeto Nakai, Kota Momose, Kotaro Yamashita, Koji Tanaka, Tomoki Makino, Kiyokazu Nakajima, Hidetoshi Eguchi, Yuichiro Doki

**Affiliations:** Department of Gastroenterological Surgery, The University of Osaka Graduate School of Medicine, 2-2 Yamadaoka, Suita-shi, Osaka, 565-0871 Japan

**Keywords:** Esophagogastric junction tumors, Esophageal involvement length, Proximal tumor margin, PET-CT, Vena cava foramen

## Abstract

**Background:**

Esophageal involvement length (EIL) is crucial to determining the surgical strategy for esophagogastric junction (EGJ) tumors. This study developed a positron emission tomography–computed tomography (PET-CT)-based method using anatomical landmarks to estimate EIL and evaluated the clinical utility of the model.

**Methods:**

We enrolled 50 patients with EGJ adenocarcinoma who underwent surgical resection. Among patients who underwent upfront surgery (Cohort 1, *n* = 24), craniocaudal distances were measured from the right and left diaphragms and vena cava foramen (VCF) to the proximal tumor margin on PET-CT images and correlated with histological EIL to establish the best-performing model. Predictive ability was validated in patients who underwent surgery after neoadjuvant chemotherapy (Cohort 2, *n* = 26).

**Results:**

In Cohort 1, among the three anatomical landmarks, VCF most strongly correlated with histological EIL (*r* = 0.81), providing the following formula: Predicted EIL (mm) = 22 + 0.3 × [Craniocaudal distance from VCF to proximal tumor margin (mm)]. The median absolute difference between the histological and predicted EILs was 4.6 mm (interquartile range, 1.6–6.3 mm), with 96% of cases (23/24) within 10 mm. Evaluation of the model in Cohort 2 found *r* = 0.81, a median absolute difference of 3.3 mm (interquartile range, 1.1–7.2 mm), and 92% of cases (24/26) within 10 mm. Predictive errors were significantly smaller in both cohorts than in endoscopic findings (Cohort 1, *P* = 0.004; Cohort 2, *P* = 0.002).

**Conclusions:**

A PET-CT-based approach using the craniocaudal distance from the VCF to the proximal margin could estimate EIL with sufficient accuracy to support surgical decision-making in clinical practice.

## Introduction

The incidence of esophagogastric junction (EGJ) adenocarcinoma has increased worldwide over the past few decades, including in Eastern Asia [1, 2]. Surgical strategies for EGJ tumors are determined primarily based on the Siewert classification, which has achieved widespread international consensus [3, 4]. However, a recent prospective multicenter study recommended determining the extent of lymph node dissection based on the esophageal involvement length (EIL) in EGJ tumors [5, 6]. EIL also serves as a critical factor in choosing between the right transthoracic and transhiatal approaches for surgery [5]. Thus, accurate preoperative assessment of EIL is crucial to determining the optimal treatment strategy for each patient.

EIL is commonly measured in clinical practice via gastrointestinal endoscopy. However, EGJ tumors are often advanced, and identification of the EGJ is difficult in cases with large or circumferential tumors, limiting accurate preoperative assessment of the EIL. Moreover, endoscopy is invasive and its reliability depends on the endoscopist’s skill. To address these issues, we previously investigated the utility of positron emission tomography–computed tomography (PET-CT) using ^18^F-fluoro-2-deoxyglucose (FDG) for novel objective assessment of the Siewert classification and EIL in EGJ tumors [7]. PET-CT is a minimally invasive modality that provides both anatomical and functional information about tumors [8]. Unlike conventional CT, ^18^F-FDG uptake allows the visualization of submucosal lesions and tumors following neoadjuvant chemotherapy (NAC) [9]. Although we previously demonstrated the utility of PET-CT in estimating EIL, one of the limitations of this method is the need to generate oblique CT slices to accurately identify the EGJ, which is somewhat subjective and time-consuming.

Therefore, a simpler and more objective method of estimating EIL using PET-CT is needed. We hypothesized that using a fixed anatomical landmark that can be identified more easily than the EGJ, such as the top of the right diaphragm (TRD), top of the left diaphragm (TLD), or vena cava foramen (VCF), may allow for efficient estimation of EIL on PET-CT images. The aim of this study was to develop a novel method for estimating EIL using a specific anatomical landmark and to evaluate its clinical utility.

## Materials and methods

### Study design and patient population

**Fig. 1:**
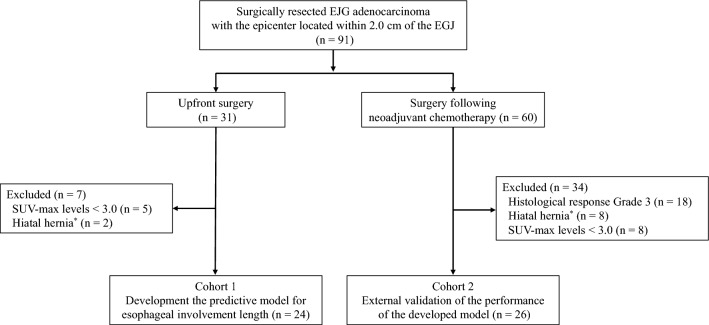
Patient flow chart. *Hiatal hernia was defined as a sac-shaped portion with gastric mucosa > 3 cm above hiatus using endoscopy

We retrospectively collected data on 91 consecutive patients with EGJ adenocarcinoma who underwent surgical resection at the University of Osaka Hospital between January 2012 and December 2023. The eligibility criteria were tumor epicenter located within 2.0 cm of the EGJ, preoperative PET-CT performed, confirmation of EIL on postoperative histological specimens, and no history of gastrectomy and/or endoscopic submucosal resection. The patient flow chart is provided in Fig. 1. The exclusion criteria were maximal standardized uptake value (SUVmax) of the primary tumor < 3.0, hiatal hernia > 3 cm above hiatus as sac-shaped portion with gastric mucosa in the endoscopy [10], or grade 3 histological response to chemotherapy. Pathological TNM staging and the pathological response to NAC were based on the 12th edition of the Japanese Classification of Esophageal Cancer [11, 12]. This study was approved by the Institutional Review Board of the University of Osaka Hospital (Approval number: 25090).

This study was conducted using two independent cohorts. The first cohort consisted of patients with EGJ tumors who underwent upfront surgery (Cohort 1) and was used to develop a predictive equation for EIL. The second cohort consisted of patients with EGJ tumors who underwent surgery following NAC (Cohort 2), to whom we applied the equation developed in Cohort 1 to evaluate its performance.

### PET protocol and image analysis

**Fig. 2:**
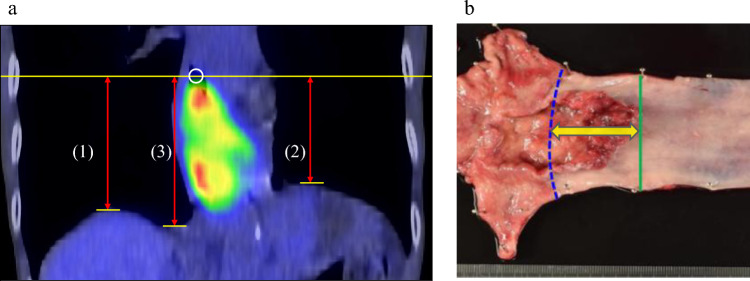
Visualization of craniocaudal distances on PET-CT (**a**) and histological EIL (**b**). a The proximal tumor margin was identified by reviewing the transaxial images and a marker placed at that location on the fused coronal image (white circle). We identified the horizontal levels of the three landmarks on the PET-CT image: (1) the top of the right diaphragm, (2) the top of the left diaphragm, and (3) the vena cava foramen. Finally, the craniocaudal distance from the level of each landmark to the level of the proximal tumor margin was measured (red double-headed arrow). **b** The blue line represents the esophagogastric junction and green line the proximal tumor margin. The yellow double-headed arrow shows the esophageal involvement length (EIL)

From January 2012 to November 2014, PET-CT imaging was performed using an integrated Gemini GXL scanner (Philips, Amsterdam, the Netherlands) with a slice thickness of 5 mm. From December 2014 to December 2023, PET-CT was performed using a Discovery PET/CT 710 system (GE Healthcare, Milwaukee, WI) with a slice thickness of 3.75 mm or a Biograph Vision 600 system (Siemens Healthineers, Erlangen, Germany) with a slice thickness of 3 mm. Tumors were assessed using Volume Viewer 2 software (GE Healthcare). First, the proximal tumor margin was identified on transaxial PET-CT images with a detection threshold of SUVmax ≥ 3.0 and marked on the fused coronal view as described previously [13]. Second, we identified the horizontal levels of the three landmarks, the TRD, TLD, and VCF, on the PET-CT images (Fig. 2a). The TRD and TLD levels were defined as the slices where the right and left diaphragmatic domes first appeared, moving cranially in the axial plane. The VCF level was defined as the slice where the inferior vena cava was clearly separated from the liver, as reported previously [14]. Finally, the craniocaudal distance from each landmark to the proximal tumor margin was measured by multiplying the number of transaxial slices between them by the slice thickness. Each landmark was set as zero, with positive values indicating that the proximal tumor margin was cranial and negative values indicating that the proximal tumor margin was caudal. In Cohort 1, PET-CT images obtained 1–4 weeks before surgery were used. In Cohort 2, PET-CT images obtained between the last administration of chemotherapy and surgery were used.

### Histological measurement of EIL

The EGJ was macroscopically defined in resected specimens as the location between the esophagus and stomach that showed a change in gastrointestinal tract diameter according to the 12th edition of the Japanese Classification of Esophageal Cancer [11, 12]. The EGJ and proximal tumor margin were determined macroscopically using surgically resected specimens, and EIL was measured based on histology (Fig. 2b). Observations of the EIL during preoperative endoscopy were the measurements described in the endoscopic report. Cases in which the EIL was not described on the report were excluded.

### Development of a predictive model for EIL

In Cohort 1, simple linear regression was performed to develop a predictive model for EIL using the craniocaudal distance from each anatomical landmark on PET-CT as the independent variable and the histological EIL as the dependent variable. A simple linear regression model was constructed in the form Y = a + bX, where a represents the intercept and b is the regression coefficient. The intercept was rounded to the nearest integer, and the coefficient was rounded to one decimal place for clinical simplicity. This model was applied to Cohort 2 and its performance evaluated by comparing the calculated EIL to the histological EIL.

### Statistical analysis

Correlations between the craniocaudal distances from each anatomical landmark on PET-CT and the histological EIL were assessed in Cohort 1 using Pearson’s correlation coefficient, with 95% confidence intervals (CIs) calculated, and a simple linear regression model was developed by the least squares method. The absolute differences of the predictive model and preoperative endoscopic findings relative to the histological EIL were compared using the Wilcoxon rank-sum test, and chi-squared was used to evaluate whether the difference was within 10 mm. In Cohort 2, correlations between the histological EIL and predicted EIL obtained using the established model were analyzed using Pearson’s correlation coefficient. *P* < 0.05 was considered significant. Statistical analysis was performed using SPSS software (version 29.0.2.0, IBM Corp., Armonk, NY, USA).

## Results

### Patient characteristics

A total of 50 patients were analyzed in this study, with 24 patients in Cohort 1 and 26 patients in Cohort 2. The background characteristics of the patients are shown in Table 1. In Cohort 1, the median tumor size was 40 mm, the median SUVmax before surgery was 7.9, and the median histological EIL was 20 mm. In Cohort 2, the median tumor size was 55 mm, the median SUVmax before surgery was 5.2, and the median histological EIL was 23 mm.

**Table 1:** Patients characteristics

Characteristics		Cohort 1 (*n* = 24)	Cohort 2 (*n* = 26)
Age (years)	Median (range)	68 (44–89)	65 (37–81)
Sex	Male	18 (75%)	21 (81%)
	Female	6 (25%)	5 (19%)
Tumor size (mm)	Median (range)	40 (18–90)	55 (25–127)
Histological type of adenocarcinoma	Differentiated	19 (80%)	17 (65%)
	Undifferentiated	5 (20%)	9 (35%)
Siewert classification	Type I	2 (8%)	3 (12%)
	Type II	22 (92%)	23 (88%)
SUV-max levels	Median (range)	7.9 (3.0–18.3)	5.2 (3.0–17.7)
Type of surgery	Subtotal esophagectomy + PG	3 (13%)	15 (57%)
	Lower esophagectomy + PG	18 (74%)	9 (35%)
	Lower esophagectomy + TG	3 (13%)	2 (8%)
(y)pT status	T1	9 (38%)	3 (12%)
	T2	1 (4%)	2 (8%)
	T3	9 (38%)	19 (73%)
	T4	5 (20%)	2 (7%)
(y)pN status	N0	12 (50%)	5 (19%)
	N1	6 (25%)	8 (31%)
	N2	4 (17%)	4 (15%)
	N3	2 (8%)	9 (35%)
Histological response	Grade 1a	–	16 (62%)
	Grade 1b	–	6 (23%)
	Grade 2	–	4 (15%)
EIL in histology (mm)	Median (range)	20 (5–40)	23 (10–45)

*SUV-max* maximal standardized uptake value, *PG* proximal gastrectomy, *TG* total gastrectomy, *EIL* length of esophageal involvement

### EIL predictive model in Cohort 1

**Fig. 3:**
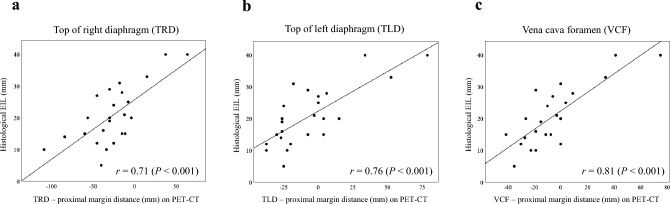
Correlations between histological EIL and craniocaudal distances on PET-CT in Cohort 1. The anatomical landmarks were the top of the right diaphragm (TRD) (**a**), the top of the left diaphragm (TLD) (**b**), and the vena cava foramen (VCF) (**c**). The TRD and TLD levels were defined as the slices where the right and left diaphragmatic domes first appeared, moving cranially in the axial plane. The VCF level was defined as the slice where the inferior vena cava was clearly separated from the liver

Figure 3 shows the correlations between the histological EIL and the craniocaudal distances from the three anatomical landmarks to the proximal tumor margin on PET-CT images; the craniocaudal distance from the VCF to the proximal tumor margin showed the strongest correlation with histological EIL (TRD: *r* = 0.71 [95% CI 0.43–0.86], *P* < 0.001; TLD: *r* = 0.76 [95% CI 0.51–0.89], *P* < 0.001; and VCF: *r* = 0.81 [95% CI 0.59–0.91], *P* < 0.001). We developed the following predictive model for EIL using the VCF as an anatomical landmark:

Predicted EIL (mm) = 22 + 0.3 × [Craniocaudal distance from the VCF to the proximal tumor margin on PET-CT (mm)].

**Fig. 4:**
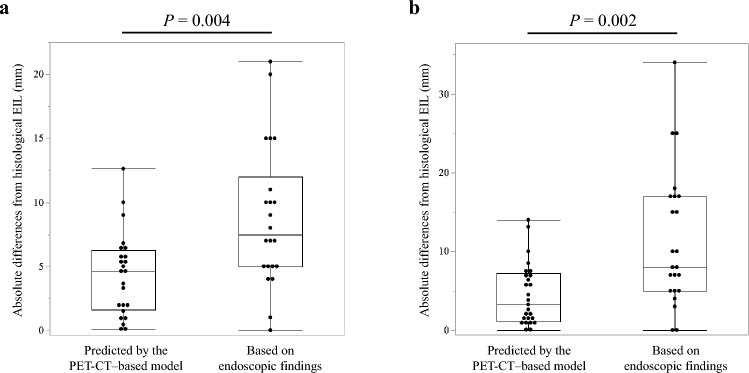
Absolute differences from histological EIL for PET-CT-based model and endoscopic findings in Cohort 1 (**a**) and Cohort 2 (**b**)

In Cohort 1, the median absolute difference between the histological EIL and the EIL predicted by this PET-CT-based model was 4.6 mm (interquartile range [IQR], 1.6–6.3 mm). The median absolute difference between the histological EIL and the EIL based on endoscopic findings before surgery was 7.5 mm (IQR, 5–12 mm; Fig. 4a). The absolute difference relative to the histological EIL was significantly smaller for the EIL predicted by the PET-CT-based model than for the EIL based on endoscopic findings (*P* = 0.004). Overall, the PET-CT-based model achieved accurate prediction in 96% of cases (23/24) with an absolute difference ≤ 10 mm, compared with 73% of cases (16/22) based on endoscopic findings. The predictive model was significantly more accurate in estimating the EIL within 10 mm (*P* = 0.029; Table 2a). Regarding the estimation of whether the EIL exceeded 3 cm, the PET-CT-based model correctly classified 96% of cases (23/24), whereas endoscopic findings correctly classified 82% of cases (18/22).

**Table 2:** Absolute differences from histological EIL between EIL predicted by the PET-CT-based model and EIL based on endoscopic findings in Cohort 1 (a) and Cohort 2 (b)

(a)				
		PET-CT-based model (*n* = 24)	Endoscopic findings (*n* = 22)	*P*
Absolute difference from histological EIL	Median (IQR), mm	4.6 (1.6–6.3)	7.5 (5–12)	0.004
	≤ 10 mm, *n* (%)	23 (96%)	16 (73%)	0.029

**(b)**				
		PET-CT-based model (*n* = 26)	Endoscopic findings (*n* = 23)	*P*
Absolute difference from histological EIL	Median (IQR), mm	3.3 (1.1–7.2)	8.0 (5–17)	0.002
	≤ 10 mm, *n* (%)	24 (92%)	14 (61%)	0.009

*PET-CT* positron emission tomography–computed tomography, *EIL* length of esophageal involvement, *IQR* interquartile range

### Validation of the model accuracy in Cohort 2

**Fig. 5:**
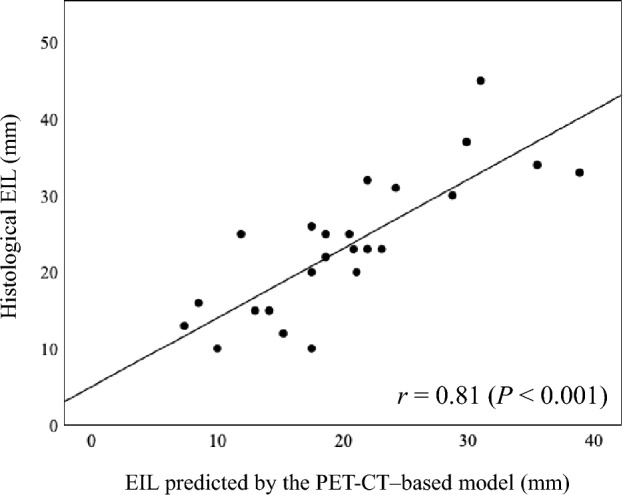
Correlation between histological EIL and PET-CT-based model-predicted EIL in Cohort 1 (**a**) and Cohort 2 (**b**)

The predictive ability of the established PET-CT-based model for EIL was validated in Cohort 2, which included patients with EGJ tumors who underwent surgery after NAC. The correlation between histological EIL and the EIL predicted by the PET-CT-based model is shown in Fig. 5. The model achieved an *r* of 0.81 (95% CI, 0.61–0.91) in Cohort 2 (*P* < 0.001).

In Cohort 2, the median absolute difference between the histological and predicted EILs was 3.3 mm (IQR, 1.1–7.2 mm), whereas the median absolute difference between the histological and endoscopic EILs was 8.0 mm (IQR, 5–17 mm; Fig. 4b). The absolute difference of the PET-CT-based model was significantly smaller than the absolute difference based on endoscopic findings (*P* = 0.002). Overall, the PET-CT-based model estimated EIL within 10 mm in 92% of cases (24/26), compared with 61% (14/23) using endoscopic findings, and was significantly more accurate (*P* = 0.009; Table 2b**)**. For determining whether EIL exceeded 3 cm, the predictive model correctly classified 88% of cases (23/26), compared with 83% of cases (19/23) using endoscopic findings.

## Discussion

In this study, we investigated the indirect predictive model for EIL using PET-CT by simply measuring the craniocaudal distances from fixed anatomical landmarks to the proximal tumor margin. Among the three fixed anatomical landmarks, the craniocaudal distance from the VCF to the proximal tumor margin had the strongest correlation with histological EIL. A predictive formula for EIL was developed using the VCF, and its performance was validated in a separate cohort. The model achieved *r* = 0.81 and demonstrated clinical applicability. Furthermore, the median absolute difference between the histological and PET-CT-based EILs was within 5 mm in both cohorts, which is smaller than the CT slice thickness and considered clinically acceptable. Compared with conventional endoscopy-based measurement of EIL, the predictive error of this model was significantly smaller, supporting its potential clinical utility.

PET-CT combines anatomical and metabolic information and is useful for diagnosing and localizing gastrointestinal cancers [15]. Regarding its diagnostic performance, PET-CT demonstrates high sensitivity of 83–98% for detecting EGJ tumors [7, 16–18]. PET-CT has been reported to be superior to CT in defining the cranial and caudal extents of esophageal tumors and to correlate well with the tumor length measured in surgical specimens [19–21]. Although useful for assessing both tumor and anatomical information in esophageal and EGJ tumors, few studies have evaluated the role of PET-CT in measuring EIL, a key factor in surgical planning [5]. We previously reported a median difference of 5 mm (IQR, 2.5–13.5 mm) from pathology when measuring EIL by PET-CT, indicating acceptable accuracy [7]. However, one of the limitations of the method was the need for technically demanding procedures, such as creating specialized oblique sections on PET-CT images.

Our novel method for estimating EIL using the VCF can be performed with standard PET-CT transaxial sections, making it simpler than previous methods. The VCF is easily identified as a fixed anatomical site where the inferior vena cava passes through the diaphragm. In contrast, TRD and TLD are influenced by respiratory motion and anatomical variation, which may have contributed to their weaker correlations with histological EIL in this study. Previous studies also suggested that the position of the proximal tumor margin relative to the VCF on CT may help predict thoracic lymph node involvement [14], supporting the VCF as a reliable landmark for EGJ tumors.

The main advantage of our method is its ability to estimate EIL even in cases where the EGJ is difficult to identify by preoperative endoscopy, such as in large or circumferential tumors. Esophagography may represent a potential alternative for preoperative evaluation of EIL. However, even when esophagography is performed in cases with large or circumferential tumors, identification of the esophagogastric junction may remain difficult, similar to endoscopic assessment. In this study, EIL could not be assessed by endoscopy in 5 of 50 patients (10%) across both cohorts prior to surgery. In addition, discrepancies > 10 mm between preoperative endoscopic findings and histological EIL were observed in 33% of cases (15/45), which may significantly affect clinical decision-making regarding surgical strategy. On the other hand, the predictive model established in this study estimated EIL significantly more accurately than preoperative endoscopic findings in both cohorts. Although endoscopy-based EIL assessment may be influenced by endoscopist experience and factors including insufflation level, our method may provide a more objective measurement because it utilizes PET-CT-derived distances based on the proximal tumor margin and the VCF, which is a consistently identifiable anatomical landmark. Moreover, after NAC, it may be more difficult to assess the extent of tumor spread using endoscopy due to fibrosis and other tissue changes. Notably, our method remained applicable even in patients who received NAC (*i.e.*, Cohort 2) by taking advantage of the high detection ability of PET-CT in residual tumor lesions [22, 23]. Although this method enables objective PET-CT-based evaluation using easily identifiable anatomical landmarks, interobserver variability was not assessed, and further validation with multiple observers is needed.

This study has several limitations. First, this was a retrospective, single-center study with a relatively small sample size. Due to the limited number of patients, the validity of the EIL prediction formula could not be evaluated in other cohorts not receiving NAC. Further investigations with larger cohorts are needed to confirm our findings and support their application in clinical practice. Second, we set a relatively high SUVmax threshold of 3.0 because distinguishing the primary tumor from surrounding inflammatory changes becomes challenging at lower uptake levels. In the results, 14% of patients (13/91) had an SUVmax < 3.0 and were therefore not evaluable for EIL assessment by PET-CT. Third, in cases with hiatal hernia, the position of the proximal tumor margin might be displaced, making it difficult to apply our method accurately. In the present analysis, such cases were excluded from the outset. Establishing an appropriate method for estimating EIL in patients with hiatal hernia remains an important challenge for future studies.

In conclusion, this study suggests that EIL can be reliably estimated on PET-CT images using the craniocaudal distance from the level of the VCF to the proximal tumor margin, even in patients who undergo surgery following NAC. Our method would be particularly useful in cases where EIL cannot be determined by conventional endoscopy. Thus, our predictive model has the potential to assist in surgical decision-making in clinical practice.

## Data Availability

The datasets generated and/or analyzed during the current study are available from the corresponding author on reasonable request.
